# Creatine Supplementation During Resistance Training Does Not Lead to Greater Bone Mineral Density in Older Humans: A Brief Meta-Analysis

**DOI:** 10.3389/fnut.2018.00027

**Published:** 2018-04-24

**Authors:** Scott C. Forbes, Philip D. Chilibeck, Darren G. Candow

**Affiliations:** ^1^Department of Physical Education, Faculty of Education, Brandon University, Brandon, MB, Canada; ^2^College of Kinesiology, University of Saskatchewan, Saskatoon, SK, Canada; ^3^Faculty of Kinesiology & Health Studies, University of Regina, Regina, SK, Canada

**Keywords:** supplements, creatine, strength, bone, health

## Abstract

Creatine supplementation during resistance training has potential beneficial effects on properties of bone in aging adults. We systematically reviewed randomized controlled trials (RCTs) investigating the effect of creatine supplementation combined with resistance training on bone mineral density (BMD) in aging adults. We searched PubMed and SPORTDiscus databases and included RCTs of ≥3 months duration that examined the combined effect of creatine and resistance training on bone mineral in adults >50 years of age or postmenopausal. Meta-analyses were performed when applicable trials were available on whole body and clinically important bone sites. Five trials met inclusion criteria with a total of 193 participants. Two of the studies reported significant benefits of creatine supplementation and resistance training compared to resistance training alone on bone. Meta-analyses revealed no greater effect of creatine and resistance training compared to resistance training alone on whole body BMD (MD: 0.00, 95% CI −0.01 to 0.01, *p* = 0.50), hip BMD (MD −0.01, 95% CI −0.02 to 0.01, *p* = 0.26), femoral neck BMD (MD 0.00, 95% CI −0.01 to 0.01, *p* = 0.71), and lumbar spine BMD (MD 0.01, 95% CI −0.01 to 0.03, *p* = 0.32). In conclusion, there is a limited number of RCTs examining the effects of creatine supplementation and resistance training on BMD in older adults. Our meta-analyses revealed no significant effect on whole body, hip, femoral neck, or lumbar spine BMD when comparing creatine and resistance training to resistance training alone. Future longer term (>12 month) trials with higher resistance training frequencies (≥3 times per week) is warranted.

## Introduction

Osteoporosis, characterized by a reduction in bone mineral and bone strength, is a leading cause of age-related disability [1]. Healthcare costs linked to treating osteoporosis are in the billions of dollars [2]; therefore, from a healthy aging and economic perspective, lifestyle interventions which decrease osteoporosis are important. It is well-established that physical activity can have a positive effect on bone health [3]. In a Cochrane systematic review which included forty-three randomized controlled trials (RCTs), aerobic, weight bearing, and resistance exercises were all able to enhance bone mineral density (BMD; [4]); however, the most effective exercise intervention for improving femoral neck (hip) BMD was progressive resistance training [4]. This is important as hip fracture results in disability, loss of functionality, and premature death [5]. While resistance training has a positive effect on bone, results are typically small (1–2% per year; [3, 6]) and may not be clinically significant for aging adults.

Creatine is a non-essential nitrogen-containing compound produced endogenously or can be exogenously consumed from foods such as red meat and seafood [7]. Creatine supplementation may have a favorable effect on aging bone (for reviews see [8, 9]). Mechanistically, creatine increases phosphorylcreatine (PCr) stores in aging muscle [10], enhancing the ability to re-synthesize adenosine triphosphate (ATP) which may lead to increased resistance-training capacity [11–13] and greater muscle mass over time in older adults (for reviews see [14–16]). An increase in muscle mass may result in greater muscle pull and stress on bone during resistance exercise leading to bone accretion [17]. We previously showed that aging males who consumed creatine (~8 g·d^−1^) during 12 weeks of supervised resistance exercise significantly increased upper limb lean tissue mass that was correlated with changes in upper limb bone mineral content. However, creatine had no effect on upper limb BMD [18].

Creatine may also have direct impact on bone turnover. Bone cells rely on the creatine kinase reaction for resynthesis of adenosine triphosphate from phosphorylcreatine and adenosine diphosphate [19]. The addition of creatine to a low serum cell culture medium increases the metabolic activity and differentiation of osteoblast cells involved in bone formation [20]. Stimulating osteoblast cell activity may enhance osteoprotegerin production, a protein which inhibits osteoclast cell activity and decreases bone resorption [21]. In a randomized double-blind study, creatine supplementation of 9 g·d^−1^ decreased urinary excretion of cross-linked n-telopeptides of Type I collagen, a marker of bone resorption, by ~3.6% compared to a 26% increase in a group of young healthy men and women during an intense 5-week resistance training program [22]. Furthermore, aging males who consumed creatine (~8 g·d^−1^) during 10–12 weeks of supervised resistance training experienced a significant decrease in bone resorption (cross-linked n-telopeptides of Type I collagen; [23]). Subsequent evidence of an anti-catabolic bone effect from creatine comes from studies involving special populations. Louis et al. [24] investigated the effects of creatine supplementation (3 g·d^−1^) without structured exercise training in young boys suffering from Duchenne (*n* = 12) and Becker (*n* = 3) muscular dystrophy, a condition which leads to accelerated bone loss. Creatine supplementation decreased the urinary excretion of cross-linked n-telopeptides of Type I collagen by 33% compared to placebo. Using a randomized, double-blind, placebo-controlled, cross-over design, Tarnopolsky et al. [29] investigated the effects of creatine supplementation (0.1 g·kg^−1^·d^−1^) in young boys (*n* = 30) with Duchenne muscular dystrophy for 4 months. Creatine supplementation attenuated the increase in urinary excretion of cross-linked n-telopeptides of Type I collagen by 19%. Results across studies indicate that creatine may have anti-catabolic effects on bone which could lead to net bone accretion over time.

In regards to bone mineral and bone strength, creatine supplementation (2% wet weight) given to growing rats (5 weeks of age; *n* = 16) for 8 weeks increased lumbar BMD and femur bone strength compared to placebo [30]. However, creatine had no effect on BMD in hypertensive male rats, a representative model of osteoporosis [31]. In postmenopausal women, long-term (1 year) creatine supplementation (~10 g·day^−1^) during resistance training increased femoral shaft subperiosteal width (indicator of bone strength) and preserved femoral neck (hip) BMD compared to placebo [26]. These results may be clinically significant because the femoral neck is often recognized as the most relevant bone site as there is significant trauma when compromised [32]. In contrast, Tarnopolsky et al. [28] found no effect from creatine supplementation (5 g·d^−1^) on BMD (whole body, hip, lumbar spine) in healthy older adults after 6 months of structured resistance-exercise training. Furthermore, 1-year of low-dose creatine supplementation (1 g·d^−1^), without resistance training, had no effect on bone health parameters in osteopenic postmenopausal women [33].

Results across studies are mixed as to whether creatine supplementation is effective for increasing bone health in older adults. Variability in bone mineral is typically high in older adults making it difficult to obtain adequate statistical power to detect differences with creatine supplementation in many individual studies. Performing a meta-analysis helps overcome these limitations by assessing large numbers of individuals simultaneously. We therefore performed a meta-analysis to assess the effect of creatine combined with resistance training compared to resistance training alone on BMD in aging adults.

## Methods used to search, select, extract, and analyze the data

Our inclusion criteria included (i) Male participants of >50 years of age or postmenopausal females, because this is the age at which BMD starts to precipitously decrease, (ii) an intervention of creatine monohydrate with resistance training, (iii) resistance training with a placebo as a comparator, (iv) measures of whole body BMD or clinically relevant bone sites (i.e., hip, spine, femoral neck, forearm BMD), and (v) RCT design with a minimum of 12 weeks in duration. We searched PubMed and SPORTDiscus databases using the key words “creatine supplementation,” “bone,” and a variety of synonyms for “resistance training” (e.g., “strength training,” “exercise”) on August 27, 2017. Searches were limited to meta-analyses, systematic reviews or RCTs with no date restriction. There were no language restrictions. Bibliographies were also reviewed. Full-text articles selected for inclusion had the following information extracted: authors, country where the study was done, date of publication, information on specific inclusion and exclusion criteria, sample size, proportion of total sample that was female and male, proportion of the total sample completing the study, mean age, details provided about the exercise intervention (frequency, intensity, time, type), duration of study, creatine dose, and administration protocol, intervention and control adherence, adverse outcomes (i.e., nausea, bloating, diarrhea, which may be associated with the experimental protocol) and primary (i.e., whole body BMD and BMD of clinically relevant sites) and secondary outcomes (included other bone health markers as well as muscle mass and strength changes). These secondary outcomes may be associated with improved bone strength.

Means and standard deviations for baseline and post-training measurements were extracted from each study for estimation of mean changes and the standard deviation of mean changes across the interventions. Change scores were calculated as pre-training mean subtracted from post-training mean. Standard deviations (SD) for the change scores were estimated from pre- and post-training standard deviations (SDpre and SDpost) using the following equation derived from the Cochrane Handbook for Systematic Reviews of Interventions [34]:

SD change score = [(SDpre)^2^ + (SDpost)^2^ – 2 × (correlation between pre- and post-scores) × SDpre × SDpost]^1/2^

In this equation we used 0.8 as the assumed correlation between pre- and post-scores.

Heterogeneity was evaluated using χ^2^ and *I*^2^ tests where heterogeneity was indicated by either χ^2^ *p*-value equal or <0.1 or *I*^2^ test value >75%. When heterogeneity was present we used a random effects model and when heterogeneity was not present we used a fixed-effects model for our meta-analysis. Mean changes and standard deviations for mean changes for individual studies and the pooled effects and their 95% confidence intervals were calculated and Forest plots were generated using Review Manager 5.3 Software. A meta-analysis was done if 3 or more studies examined the same outcome measure. Significance was set at *p* ≤ 0.05. Risk of bias was also assessed on criteria derived from the *Cochrane Handbook for Systematic Reviews of Interventions* [34], including random sequence generation, allocation concealment, blinding of participants, blinding of outcome assessment or personnel, incomplete outcome data, and selective reporting.

## Results

### Study characteristics

**Figure 1:**
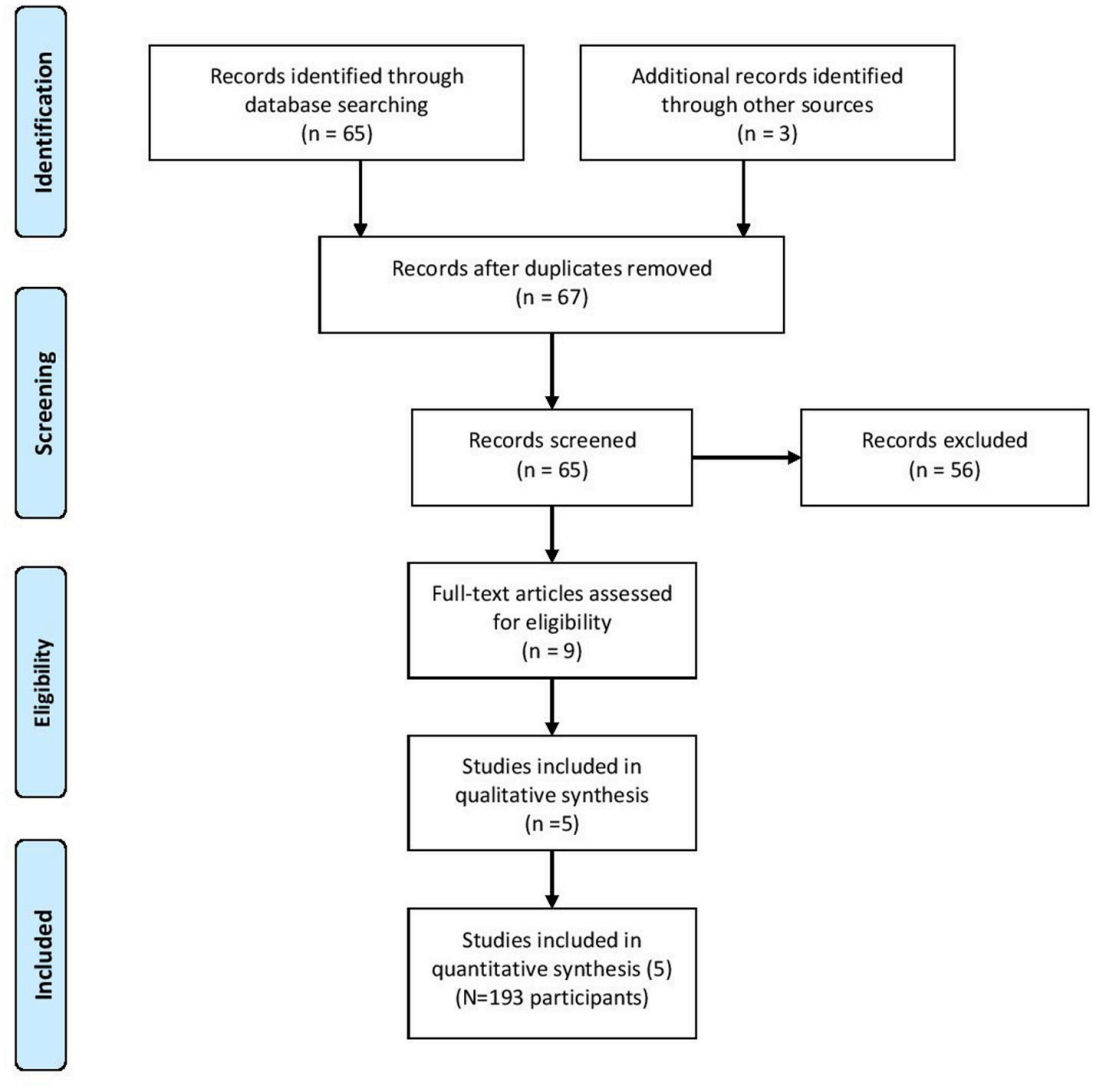
PRISMA flow chart.

Sixty-seven citations, excluding duplicate entries, were identified as potentially relevant. After the initial screening of title and abstracts, nine full-text articles were retrieved for detailed review (Figure 1). Following full text review, 5 trials met the inclusion criteria with a total of 193 participants. Among these studies there was a large amount of heterogeneity with regards to the duration of the intervention (12 weeks to 1 year), frequency of exercise per muscle per week (1.5–3 times per week), and participant characteristics (Table 1). Two studies reported beneficial effects on at least one marker of bone biology in the creatine plus resistance training group compared to resistance training and placebo [18, 26], while the other 3 reported no effects [25, 27, 28].

**Table 1:** Study characteristics and outcomes of research examining the influence of creatine with a resistance training program on bone.

**References**	**Study Population**	**Intervention**	**Duration**	**Outcomes**
[25]	*N* = 32; Healthy, non-athletic men and women between 60–80 yrs.	RCT: PLA + RT or CR + RT. Creatine group received 5 g/day. PLA received 5 g/day of maltodextrin. RT was preformed 3x/wk for 60 min and progressed on an individual basis. Muscle groups (i.e., upper and lower body) alternated between training days; 1.5x/wk per muscle group.	12 wks	CR + RT ↑ lean mass compared to PLA + RT. ↔ 10 RM in bench press or leg press, body composition, BMD, and BMC of all assessed sites between groups.
[26]	*N* = 33; Postmenopausal women; Age: 57 ± 6 yrs	RCT: PLA + RT or CR + RT. Creatine group received 0.1 g/kg/day (0.05 g/kg provided immediately before and 0.05 g/kg after training on training days and with two meals on non-training days). PLA received corn starch maltodextrin. RT was preformed 3x/wk.	12 mths	CR attenuated rate of femoral neck BMD loss compared to PLA and ↑ femoral shaft subperiosteal width. CR ↑ bench press strength more than PLA. ↔ between groups on all other outcome measures including muscle mass and muscle thickness.
[27]	*N* = 60; older vulnerable women (age: 66 yrs)	RCT: PLA, CR, PLA + RT, CR + RT. Creatine was provided 20 g/day for 5 days followed by 5 g/day for the remaining 24 wks. RT = 2x/wk.	24 wks	CR + RT ↑ appendicular lean mass accrual compared to all other groups. ↔ fat mass, bone mass, and serum bone markers between groups.
[18]	*N* = 29; older men (71 yrs)	RCT; CR + RT, PLA + RT. Creatine was provided 0.3 g/kg/day for 5 days and then 0.7 g/kg/day for the remaining. RT was performed 3x/wk	12 wks	↑ arm BMC greater in the CR group compared to PLA. ↑ in leg press strength in the CR group compared to PLA, ↔ in chest press strength between groups ↔ between groups for whole-body and leg BMD (sig. main effect for time).
[28]	*N* = 39; Older men and women (65–85 yrs)	RCT; CR + CLA + RT, PLA + RT. Creatine was provided 5 g/day. RT = 2x/wk	6 mths	↔ between groups for total BMD, hip, and Lumbar BMD. CR + RT ↑ FFM and isokinetic strength compared to PLA.

*RCT, randomized controlled trial; PLA, placebo; RT, resistance training; CR, creatine; RM, repetition maximum; BMD, bone mineral density; BMC, bone mineral content; ↑Significant greater; ↔No difference between conditions; wk, weeks; yrs, years; g, grams; kg, kilograms*.

### Compliance

All studies reporting on compliance were comparable between the creatine and placebo conditions [18, 25–27]. Average compliance to the exercise interventions ranged from 75 to 95.3% [18, 25–27]. Only one study did not report compliance, however, compliance was monitored [28]. In addition, only one study reported compliance to the supplement and again was comparable between groups (CR = 79% and PLA = 78%; [26]).

### Adverse events

Four studies [18, 25, 27, 28] reported no adverse effects of the experimental intervention. Gualanao et al. [27] further assessed clinical renal and hepatic blood markers and found no effect on urea, creatinine, or creatine kinase. Chilibeck et al. [26] was the only study to report adverse effects that were considered “possibly” or “probably” related to creatine supplementation. Seven participants reported adverse events compared to only 4 in the placebo group. Five participants reported mild gastrointestinal adverse events (i.e., constipation, diarrhea, heartburn, and irritable bowel, and nausea) and two reported muscle cramps (mild to moderate). When both gastrointestinal and musculoskeletal adverse events were grouped, the creatine group had a significantly (*p* < 0.05) higher number of events compared to placebo. Of note, there were no serious adverse events reported from creatine supplementation.

## Risk of bias

**Figure 2:**
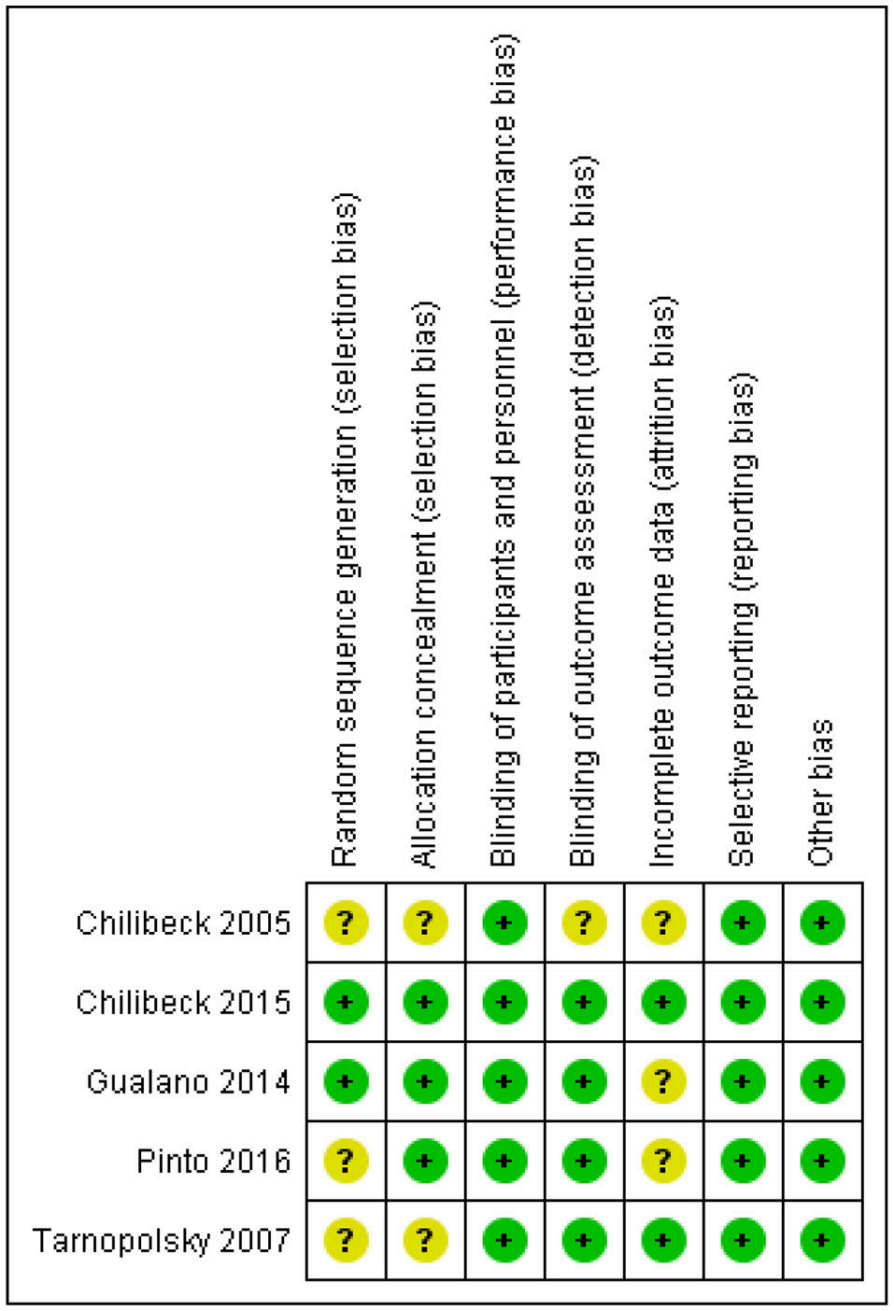
Risk of bias assessment.

One study had a rigorous experimental design and was considered low risk [26]. Four trials [18, 25, 27, 28] were either unclear or did not report on various other potential biases and were considered to be moderate risk (Figure 2).

## Meta-analyses

**Figure 3:**
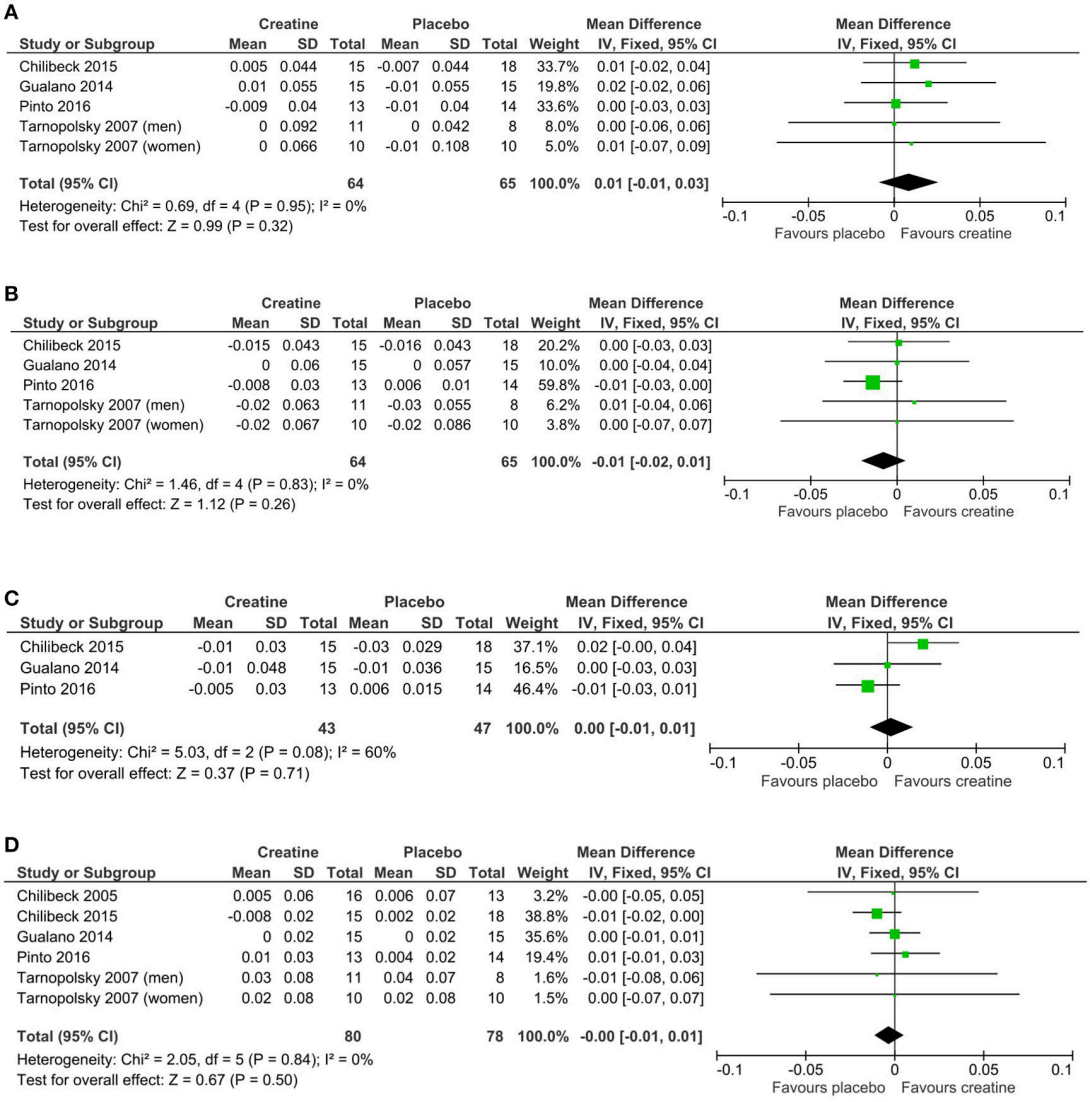
Meta-analyses of creatine and resistance training studies on **(A)** lumbar spine, **(B)** hip, **(C)** femoral neck, and **(D)** whole body bone mineral density.

Mean changes and standard deviations for mean changes for individual studies and pooled effects and their 95% confidence intervals are presented along with Forest plots in Figures 3A–D. There was no greater effect from creatine supplementation during resistance training compared to resistance training and placebo for whole body (*p* = 0.50), lumbar spine (*p* = 0.32), hip (*p* = 0.26), and femoral neck (*p* = 0.71) BMD. No studies reported changes in forearm BMD.

## Discussion

There is evidence from individual studies that creatine supplementation and resistance training increase properties of aging bone (Table 1), however, our meta-analysis failed to show a greater effect from creatine on BMD compared to placebo. The five studies included in our review had relatively low sample sizes and therefore lacked statistical power individually. Performing a meta-analysis enabled us to determine with greater statistical power whether creatine has a beneficial effect on BMD in aging adults. Despite the greater statistical power, our meta-analysis revealed that creatine in combination with resistance training provided no evidence of benefit on whole body, lumbar spine, hip, or femoral neck BMD compared to resistance training alone.

Mechanistically, creatine supplementation can potentially influence bone turnover both indirectly and directly. Indirectly, creatine can enhance muscle mass and strength adaptations following resistance training [16] and thus increase the pull on bone [18]. In aging males (65–71 years, *n* = 13–23), creatine supplementation (8 g·d^−1^) during 12 weeks of resistance training (3 days/week) increased upper limb bone mineral content [18]. These changes in arm bone mineral content were significantly correlated with changes in arm lean tissue mass [18]. With respect to the current meta-analysis, all of the included studies found either an enhanced muscle mass [25, 27, 28] or strength [18, 26, 28] in the creatine group compared to placebo. However, based on the meta-analysis, these adaptations did not translate to greater BMD. Creatine may also have direct impact on bone turnover. Bone cells rely on adenosine triphosphate rephosphorylation via the creatine kinase reaction [19]. Creatine added to a low serum culture medium increased metabolic activity and differentiation of osteoblasts cells [20], which inhibit osteoclast activity and decrease bone resorption [21]. In human studies, urinary excretion of cross-linked n-telopeptides of Type I collagen decreased following creatine consumption [22, 23], suggesting possible anti-catabolic bone effects. However, these biological plausible mechanisms did not result in improved BMD.

In regards to bone mineral and strength there is evidence from individual studies suggesting a possible beneficial effect. Creatine supplementation (10 g·d^−1^) during 12 months of resistance training (3 days/week) decreased femoral neck bone loss and increased femoral shaft subperiosteal width (indicator of bone strength) in postmenopausal women compared to placebo [26]. In aging males (65–71 years, *n* = 13–23), creatine supplementation (8 g·d^−1^) during 10–12 weeks of whole-body resistance-exercise training (3 days/week) increased upper limb bone mineral content compared to older men on placebo during training [18]. Three studies utilized absolute dosing (5g/d; [25, 27, 28]) while two studies utilized relative dosing (0.1g/kg/d; [18, 26]). Both studies using relative dosing found significant effects of creatine on bone mineral. Another methodological difference between studies finding a positive effect compared to no effect of creatine was frequency of training (3 days vs. 1.5–2 days/week/muscle group). Studies utilizing higher frequency of training found positive effects. Future research is required to directly compare higher and lower frequencies with and without creatinine. Other methodological differences included participants' characteristics (osteopenic, postmenopausal, older healthy adults) which may have impacted the findings of the meta-analysis. Lastly, study duration ranged from 12 to 52 weeks. A potential limitation of our inclusion criteria was including studies of 3 months duration. Chilibeck et al. [18], found an effect of creatine and resistance training on bone mineral content after 3 months; however, bone turnover is a relatively slow process and may require 9 months to detect robust changes [26, 35]. The only study investigating BMD in older adults with creatine and resistance over 9 months found positive effects on bone compared to placebo [26]. These methodological differences may have masked the potential effect of creatine on bone mineral in older adults. As such, future research is warranted to further elucidate the optimal dose, training frequency, and study duration.

A potential limitation of the present meta-analysis is the high risk of bias (Figure 2). Only one study demonstrated a low risk of bias [18]. These biases may have led to the equivocal findings. Future research utilizing rigorous methodology is recommended.

## Conclusion

There is a limited number of RCTs examining the effects of creatine supplementation and resistance training on bone in older adults. Our meta-analyses revealed no effect on whole body, hip, femoral neck, or lumbar spine BMD when comparing creatine and resistance training compared to resistance training alone. Interestingly, only the studies which used a resistance training frequency of 3 times per muscle group per week in combination with a relative dosing of creatine supplementation found a beneficial effect compared to resistance training alone. Future longer term studies (>12 months) with rigorous methodology utilizing a higher training frequency with and without creatine may be warranted.

## Author contributions

SF, DC, PC: contributed to the conception, design, analysis, interpretation of the work, as well as drafted, revised, and edited the manuscript.

### Conflict of interest statement

The authors declare that the research was conducted in the absence of any commercial or financial relationships that could be construed as a potential conflict of interest.
